# Factors associated with recent and regular non-use of dental services by students from a university in southeastern Brazil: a cross-sectional study

**DOI:** 10.1186/s12903-022-02648-7

**Published:** 2022-12-15

**Authors:** Rafaela de Oliveira Cunha, Isabel Cristina Gonçalves Leite

**Affiliations:** 1 Postgraduate Program in Public Health, Federal University of Juiz de Fora, José Lourenço Kelmer, São Pedro, Juiz de Fora, Minas Gerais Brazil; 2 School of Medicine, Federal University of Juiz de Fora, Eugênio do Nascimento, Dom Bosco, Juiz de Fora, Minas Gerais Brazil

**Keywords:** Access to health services, Oral health services, Oral health, Young adults

## Abstract

**Background:**

Lack of use of dental services can be a risk factor for oral health. In addition to recent visits to dental services, it is important to assess the regularity of use of these services, as well as the motivations for visiting the dentist. There is a gap in literature studies on the patterns of use of oral health services by the young university students. The goal of this study was to assess the factors associated with recent and regular non-use of dental services by young university students, using the Andersen model as a reference.

**Methods:**

This was a cross-sectional study with 477 university students between 18 and 24 years old, carried out as a web survey, through which predisposing, enabling and need variables were collected, according to the model proposed by Andersen, to test the factors associated with recent and regular non-use of dental services. Bivariate analyses and robust Poisson regression were performed, with estimation of crude and adjusted prevalence ratios, using confidence intervals of 95%. The variables with *p* < 0.05 remained in the final model.

**Results:**

The prevalence of recent non-use was of 19.5% (95% CI 16.0–23.3%), and of regular non-use, of 53.5% (95% CI 48.9–58.0%). After the adjusted analysis, the following were found to be associated with the outcome of recent non-use: type of service used (PR = 0.91; 95% CI 0.85–0.98) and perceived need for dental treatment (PR = 0.98; 95% CI 0.97–0.99); and the following variables were associated with regular non-use: father’s level of education (PR = 0.86; 95% CI 0.78–0.96), area of study (PR = 1.08; 95% CI 1.02–1.15), reason for last dental appointment (PR = 0.81; 95% CI 0.75–0.88), use of dental services throughout childhood (PR = 0.92; 95% CI 0.86–0.97), self-perceived oral health (PR = 0.86; 95% CI 0.76–0.88), and toothaches over the last 2 years (PR = 0.93; 95% CI 0.87–0.99).

**Conclusion:**

The motivation for young university students to use dental services are curative treatment needs, not prevention. The results point to the need to implement health prevention and promotion policies in higher education institutions and to expand access to dental services for this young population.

## Background

The use of health services is at the core of the functioning of health systems [1]. However, recent studies point to the existence of great inequities in the use of dental services by the general population [2–4].

Many factors can act facilitating or restricting the use of health services by individuals [5]. According to the theoretical model proposed by Andersen [6] and widely used in literature [7], the use of these services results from the interaction of individual factors, characteristics of the health system, and the social context and past experience of using the services.

In addition to the pattern of use of dental health services, the literature has pointed to the importance of assessing the regularity of the habit of visiting the dentist, identifying those individuals who consult this professional only in cases of pain or curative treatment and those who seek them out for preventive treatment [8].

Different population groups have been evaluated regarding the use of dental services. However, most epidemiological studies follow the parameters recommended by the World Health Organization (WHO) and the age index does not include young people aged between 18 and 24 years old [9]. The university environment brings together many individuals in this age group, who have relatively similar characteristics but, at the same time, have diverse experiences and lives [10]. These young people are characterized as individuals undergoing constant behavioral and lifestyle changes, which may interfere with both general health standards [11] and oral health [12].

A study conducted in southern Brazil with the university population revealed inequalities related to socioeconomic factors in the regular use of dental services and a lower use among university students with worse oral health conditions. In addition, students reported using the service more to solve oral health problems and not on a regular basis to prevent aggravations [8]. However, there is still little studies about the patterns of dental service use among the young university population in the literature. Considering the specificities of this population, more studies are needed that aim to understand the factors associated with non-use of dental services, identifying the portions of the population with greater difficulty in access and thus, assisting in the development of public health policies aimed at specific populations in an equitable manner [13, 14].

Thus, this study aims to assess the factors associated with recent and regular non-use of dental services by young university students, using the Andersen model as a reference [6].

## Methods

### Study design and participants

This was a cross-sectional study carried out through a census of university students admitted in 2021 to the Federal University of Juiz de Fora (UFJF). The public university is based in the city of Juiz de Fora (MG), and also has an advanced campus in Governador Valadares (MG). In 2021, 2480 students entered the university in the first semester and 1501 students in the second semester, for a total of 3981 students entered in presencial courses.

The study included students entering undergraduate courses at UFJF in 2021 aged between 18 and 24 years. Students who did not respond to the survey questionnaire sent by e-mail after three attempts at contact were considered as sample losses.

The parameters used to calculate sample size for finite populations were: a 45% prevalence in the regular non-use for this type of population [8], a 95% confidence interval and sampling error of 5%, resulting in a total of 347 individuals.

The study was approved by the Human Research Ethics Committee of the UFJF, under protocol number 4.617.665.

### Data collection

Data collection was carried out during the COVID-19 pandemic, between May and November 2021, a period in which emergency remote teaching had been adopted by UFJF. Thus, a survey was created on the Google Forms platform was made available via email to all students admitted in 2021; their access was conditioned to signing the Informed Consent Form.

The survey included objective questions about socio-demographic and socioeconomic characteristics, information related to the student’s major, their admission to the University and questions regarding the use of dental services and oral health status. A pilot study was carried out to test the instrument prior to data collection and allowed estimating a response time of around four minutes. Based on the pilot study, some changes were made to the survey used in order to improve understanding and interpretation of the questions.

### Variables

Two outcomes that characterize the use of dental services by the studied population were investigated. The first outcome was the recent non-use of dental services, assessed through the question “Have you accessed dental care in the last 2 years?”, with “yes” and “no” as possible answers, and non-recent use assigned to the answer “no”. The time parameter for recent use of dental services adopted in this study was 2 years, and not 12 months, as has been advocated in most studies. The choice of this interval stems from the pandemic situation caused by COVID-19, which limited patients access to dental care worldwide for a long period of time.

The World Health Organization (WHO) and the Pan American Health Organization (PAHO) conducted a survey in 128 countries to establish the degree by which attention to Noncommunicable Diseases (NCDs) was disrupted by the impact of the COVID-19 pandemic. The effect of the COVID-19 pandemic on the number of clinical dental consultations has also been examined, and a significant decrease observed [15–17]. A study in Brazil compared the mean number of clinical dental consultations in the period March-July in the years 2015–2019 with the same period in 2020, and a decrease of 65.6% in dental consultations was found [18].

The second outcome investigated was the regular non-use of dental services, measured through the question: “Which of the statements below describes your access to dental care?”, with the following answer options: “I never go to the dentist”; “I go to the dentist when I have a problem or when I know I need to have something treated”; “I go to the dentist occasionally, whether or not I have some kind of problem”; and “I go to the dentist regularly.” [19] The first two answer choices were assigned to regular non-use.

**Fig. 1:**
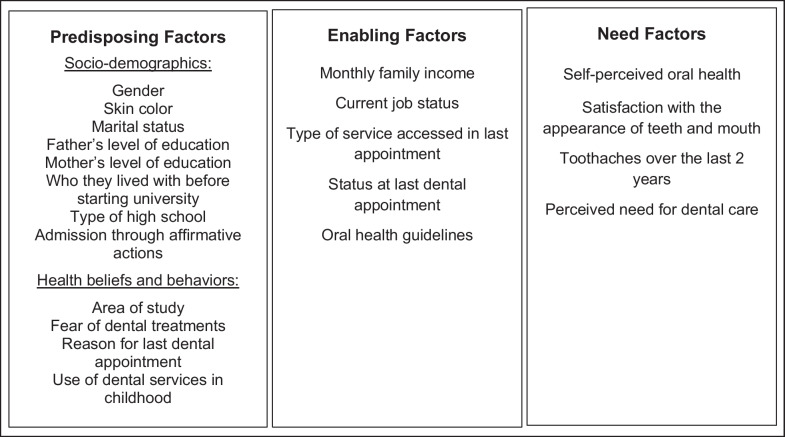
Independent variables grouped according to Andersen’s Behavioral Model [6]. Source: The authors.

The independent variables were grouped into three categories, according to the theoretical model for determining the use of health services proposed by Andersen [6] (Fig. 1). This model addresses the complexity of health services use in a comprehensive way, categorizing the determinants of service use into predisposing factors (that make the individual more or less susceptible to using health services), enabling factors (related to the possibility of accessing the service) and factors related to the individual’s need. Predisposing factors, in turn, are divided into demographics, social structure and health beliefs.

In this study, predisposing factors included: gender (cisgender woman; cisgender man; transgender, agender or nonbinary), skin color (black or brown; white), marital status (single or married/Common-law marriage), Father’s level of education and mother’s level of education (did not study/did not finish elementary school; elementary school/did not finish high school; high school degree/did not finish undergrad degree; university degree/graduate degree), living situation before starting University (alone/with friends/with partner or With family), type of high school (public or private), admission through affirmative actions (yes or no), area of study (Biological Sciences/Health Sciences or Exact and Earth Sciences/Engineering/Agricultural Sciences/Human, Sciences/Applied Social Sciences/Linguistics, Letters and Arts), reason for last dental appointment (symptomatic or prevention), use of dental services in childhood (no or yes). The enabling factors were: monthly family income (up to 1.5 minimum wage; from 1.5 to 3 minimum wages; from 3 to 6 minimum wages; over 6 minimum wages), Current job status (working or not working), type of service accessed in last appointment (public or private), status at last dental appointment (very bad/bad/regular or very good/good), oral health guidelines (no or yes). And the factors related to the needs of the individuals were: self-perceived oral health (bad/regular; or excellent/good/very good), satisfaction with the appearance of teeth and mouth (very unhappy/unhappy/neither happy nor unhappy; or very happy/happy), toothaches over the last 2 years (yes or no), perceived need for dental care (yes or no).

### Data analysis

Data analysis was conducted using the Statistical Package for the Social Sciences (SPSS) software, version 20.0 for Windows. Initially, descriptive analyses were performed using absolute and relative frequencies. The association of dependent and independent variables was investigated through bivariate analysis and robust Poisson regression with estimation of crude and adjusted prevalence ratios, using confidence intervals of 95%. The associated independent variables with a value of p ≤ 0.05 entered the multiple model, while the variables with a value of *p* < 0.05 remained in the final model.

## Results

Of the 3,981 entering undergraduates, 581 responded to the survey questionnaire, which corresponded to a response rate of 14.6%. Of these, 477 met the inclusion criteria of the study.

Therefore, the final sample consisted of 477 university students aged between 18 and 24 years old. The majority were cisgender women (66.9%), aged between 18 and 19 years (74.0%) and white (64.5%). Most students attended public high schools (51.8%), had a monthly family income between 1.5 and 3 minimum wages (26.2%), lived with their parents and/or other family members (80.5%), and did not work (81.8%). In addition, most reported that their father or mother had a high school degree (31.0% and 29.1% respectively). Regarding their area of study, most participants were enrolled in courses in the health sciences (41.7%). Of the participants, 87.8% self-rated their oral health as excellent, very good or good, and 57.4% were happy with the appearance of their mouth and teeth. Despite this, 43.4% reported having had a toothache in the last two years and 59.3% reported needing dental treatment. Most participants used private dental health services (86.3%).

Table 1 presents the demographic, socioeconomic and oral health characteristics of the young university students grouped according to Andersen’s theoretical model^6^ and distributed by the investigated outcomes. The prevalence of recent non-use was 19.5% (95% CI 16.0–23.3%) and of regular non-use was 53.5% (95% CI 48.9–58.0%).

**Table 1:** Demographic, socioeconomic and oral health characteristics of university students distributed by investigated outcome. *Source*: Research data

Variable	Recent use of dental services				Regular use of dental services			
	No		Yes		No		Yes	
	n	%	n	%	n	%	n	%
**Predisposing factors**								
*Socio-demographic characteristics*								
Age group								
18 and 19 years old	70	75.3	283	73.7	190	74.5	163	73.4
20 years old and over	23	24.7	101	26.3	65	25.5	59	26.6
Self-reported skin color								
Black or brown	38	40.9	131	34.1	94	36.9	75	33.8
White	55	59.1	253	65.9	161	63.1	147	66.2
Gender								
Cisgender woman	52	55.9	267	69.5	174	68.2	145	65.3
Cisgender man	37	39.8	113	29.4	76	29.8	74	33.3
Transgender, agender or nonbinary	4	4.3	4	1	5	2	3	1.4
Marital status								
Single	92	98.9	380	99	251	98.4	221	99.5
Married/Common-law marriage	1	1.1	4	1	4	1.6	1	0.5
Father’s level of education								
Did not study/Did not finish elementary school	22	25.6	66	17.6	55	22.4	31	15.2
Elementary school/Did not finish high school	9	10.5	48	12.8	36	14.7	21	9.7
High school degree/Did not finish undergrad degree	39	45.3	138	36.7	98	40	79	36.4
University degree/Graduate degree	16	18.6	124	33	56	22.9	84	38.7
Mother’s level of education								
Did not study/Did not finish elementary school	14	15.2	42	11	36	14.2	20	9
Elementary school/Did not finish high school	11	12	33	8.6	28	11	16	7.2
High school degree/Did not finish undergrad degree	34	37	128	33.4	87	34.3	75	33.9
University degree/Graduate degree	33	35.9	180	47	103	40.6	110	49.8
Living situation before starting University								
Alone/With friends/With partner	4	4.3	12	3.1	11	4.3	5	2.3
With family	89	95.7	372	96.9	244	95.7	217	97.7
Type of high school institution								
Public	58	62.4	189	49.2	145	56.9	102	45.9
Private	35	37.6	195	50.8	110	43.1	120	54.1
Admitted to UFJF through affirmative actions								
Yes	47	50.5	158	41.1	118	46.3	87	39.2
No	46	49.5	226	58.9	137	53.7	135	60.8
*Health beliefs and behaviors*								
Area of study								
Biological Sciences/Health Sciences	33	35.5	177	46.1	92	36.1	118	53.2
Exact and Earth Sciences/Engineering/Agricultural Sciences/Human Sciences/Applied Social Sciences/Linguistics, Letters and Arts	60	64.5	207	53.9	163	63.9	104	46.8
Fear of dental treatments								
Yes	18	19.4	75	19.5	64	25.1	29	13.1
No	75	80.6	309	80.5	191	74.9	193	86.9
Reason for last dental appointment in the last 2 years								
Symptomatic	12	57.1	85	22.1	76	39.6	21	9.9
Prevention	9	42.9	299	77.9	116	60.4	192	90.1
Use of dental services in childhood								
No	52	55.9	155	40.4	137	53.7	70	31.5
Yes	41	44.1	229	59.6	118	46.3	152	68.5
*Enabling Factors*								
Monthly family income								
Up to 1.5 minimum wage	29	31.2	72	18.8	66	25.9	35	15.8
From 1.5 to 3 minimum wages	28	30.1	97	25.3	72	28.2	53	23.9
From 3 to 6 minimum wages	21	22.6	121	31.5	72	28.2	70	31.5
Over 6 minimum wages	15	16.1	94	24.5	45	17.6	64	28.8
Job status								
Working	19	20.4	68	17.7	50	19.6	37	16.7
Not working	74	79.6	316	82.3	205	80.4	185	83.3
Has received guidance on oral health promotion from a professional								
No	9	9.7	24	6.2	21	8.2	12	5.4
Yes	84	90.3	360	93.8	234	91.8	210	94.6
Type of service used in last dental appointment								
Public	33	36.3	32	8.3	42	16.6	23	10.4
Private	58	63.7	352	91.7	211	83.4	199	89.6
Status at last dental appointment								
Very bad/Bad/Regular	16	17.2	22	5.7	30	11.8	8	3.6
Very good/Good	77	82.8	362	94.3	225	88.2	214	96.4
*Need factors*								
Self-perceived oral health								
Bad/Regular	24	25.8	34	8.9	47	18.4	11	5
Excellent/Good/Very good	69	74.2	350	91.1	208	81.6	211	95
Satisfaction with appearance of teeth and mouth								
Very unhappy/Unhappy/Neither happy nor unhappy	51	54.8	152	39.6	124	48.6	79	35.6
Very happy/Happy	42	45.2	232	60.4	131	51.4	143	64.4
Toothaches over the last 2 years								
Yes	37	39.8	170	44.3	133	52.2	74	33.3
No	56	60.2	214	55.7	122	47.8	148	66.7
Self-perceived need for dental treatment								
Yes	71	76.3	212	55.2	168	65.9	115	51.8
No	22	23.7	172	44.8	87	34.1	107	48.2

In the crude analysis, the following variables belonging to the predisposing factors showed significant differences when associated with recent non-use of dental services: gender, father’s level of education, type of high school institution, reason for last dental appointment and use of dental services in childhood. Among the variables listed as enabling factors, the following stand out: monthly family income, type of service used and status at last dental appointment. And of those classified as need factors: self-perception of oral health, satisfaction with the appearance of teeth and mouth, and perceived need for dental treatment. After the adjusted analysis, the following variables remained associated with recent non-use: type of service used and perceived need for dental treatment.

In the crude analysis phase, regular non-use of dental services was associated with the following variables of predisposing factors: father’s level of education, type of high school institution, area of study, fear of dental treatment, reason for last dental appointment and use of dental services in childhood. Among the variables related to enabling factors, this outcome was associated with the following: monthly family income, type of service used and status at last dental appointment; and the following were related to need factors: self-perception of oral health, satisfaction with the appearance of teeth and mouth, toothaches over the last 2 years, and perceived need for dental treatment. After the adjusted analysis, the following variables remained associated with regular non-use: father’s level of education, area of study, reason for last dental appointment, use of dental services in childhood, self-perception of oral health, and toothaches over the last 2 years. Crude and adjusted prevalence ratios for recent and regular non-use of dental services are presented in Tables 2 and 3, respectively.

**Table 2:** Crude and adjusted prevalence ratios for recent non-use of dental services. *Source*: Research data

Variable	%	Crude PR (95%CI)	*p*^a^	Block-adjusted PR (95%CI)	*p*^b^	Adjusted PR-final model (95%CI)	*p*^b^
**Block 1-Predisposing factors**							
*Socio-demographic characteristics*							
Gender			0.009				
Cisgender woman	16.3	1.22 (0.97–1.54)					
Cisgender man	24.7	1.17 (0.92–1.48)					
Transgender, agender or nonbinary	50.0	1					
Father’s level of education			0.032				
Did not study/Did not finish elementary school	25.0	0.93 (0.87–0.98)					
Elementary school/Did not finish high school	15.8	0.98 (0.92–1.04)					
High school degree/Did not finish undergrad degree	22.0	0.94 (0.90–0.99)					
University degree/Graduate degree	11.4	1					
Type of high school institution			0.028				
Public	23.5	1.54 (1.06–2.25)					
Private	15.2	1					
*Health beliefs and behaviors*							
Reason for last dental appointment in the last 2 years			< 0.001		0.031		
Symptomatic	12.4	4.23 (1.84–9.74)		0.96 (0.93–0.99)			
Prevention	2.9	1		1			
Use of dental services in childhood			0.007				
No	25.1	1.65 (1.15–2.39)					
Yes	15.2	1					
*Block 2-Enabling factors*							
Monthly family income			0.015				
Up to 1.5 minimum wage	28.7	0.92 (0.86–0.98)					
From 1.5 to 3 minimum wages	22.4	0.95 (0.90–1.01)					
From 3 to 6 minimum wages	14.8	0.99 (0.95–1.04)					
Over 6 minimum wages	13.8	1					
Type of service used in last dental appointment			< 0.001		< 0.001		0.007
Public	50.8	3.59 (2.56–5.03)		0.81 (0.75–0.88)		0.91 (0.85–0.98)	
Private	14.1	1		1		1	
Status at last dental appointment			< 0.001		0.017		
Very bad/Bad/Regular	42.1	2,40 (1.57–3.67)		0.89 (0.81–0.98)			
Very good/Good	17.5	1		1			
*Block 3-Need factors*							
Self-perceived oral health			< 0.001		0.005		
Bad/Regular	41.4	2.51 (1.73–3.65)		0.88 (0.81–0.96)			
Excellent/Good/Very good	16.5	1		1			
Satisfaction with appearance of teeth and mouth			0.008				
Very unhappy/Unhappy/Neither happy nor unhappy	25.1	1.64 (1.14–2.36)					
Very happy/Happy	15.3	1					
Self-perceived need for dental treatment			< 0.001		0.009		0.018
Yes	25.1	2.21 (1.42–3.44)		0.95 (0.91–0.99)		0.98 (0.97–0.99)	
No	11.3	1		1		1	

*PR* prevalence ratio, *95%CI* Confidence interval

^a^Pearson’s chi-square test

^b^Poisson regression with robust variance

**Table 3:** Crude and adjusted prevalence ratios for regular non-use of dental services. *Source*: Research data

Variable	%	Crude PR (95%CI)	*p* ^a^	Block-adjusted PR (95%CI)	*p* ^b^	Adjusted PR-final model (95%CI)	*p* ^b^
Block 1-Predisposing Factors							
Socio-demographic Characteristics							
Father’s level of education			0.001		0.019		0.042
Did not study/Did not finish elementary school	62.5	0.86 (0.79–0.94)		0.91 (0.83–0.99)		0.92 (0.84–1.02)	
Elementary school/Did not finish high school	63.2	0.85 (0.77–0.95)		0.86 (0.78–0.95)		0.86 (0.78–0.96)	
High school degree/Did not finish undergrad degree	55.4	0.90 (0.84–0.97)		0.93 (0.87–1.00)		0.94 (0.88–1.01)	
University degree/Graduate degree	40	1		1		1	
Type of high school education			0.022				
Public	58.7	1.23 (1.03–1.46)					
Private	47.8	1					
Health beliefs and behaviors							
Area of study			< 0.001		0.002		0.006
Biological Sciences/Health Sciences	43.8	0.72 (0.60–0.86)		1.09 (1.03–1.16)		1.08 (1.02–1.15)	
Exact and Earth Sciences/Engineering/Agricultural Sciences/Human Sciences/Applied Social Sciences/Linguistics, Letters and Arts	61.0	1		1		1	
Fear of dental treatments			0.001		0.039		
Yes	68.8	1.38 (1.17–1.64)		0.92 (0.85–0.99)			
No	49.7	1		1			
Reason for last dental appointment in the last 2 years			< 0.001		< 0.001		< 0.001
Symptomatic	78.4	2.08 (1.74–2.48)		0.79 (0.72–0.85)		0.81 (0.75–0.88)	
Prevention	37.7	1		1		1	
Use of dental services in childhood			< 0.001		0.001		0.006
No	66.2	1.51 (1.28–1.79)		0.90 (0.85–0.96)		0.92 (0.86–0.97)	
Yes	43.7	1		1		1	
Block 2-Enabling Factors							
Monthly family income			0.004		0.024		
Up to 1.5 minimum wage	65.3	0.85 (0.77–0.93)		0.87 (0.79–0.95)			
From 1.5 to 3 minimum wages	57.6	0.90 (0.82–0.98)		0.91 (0.84–0.99)			
From 3 to 6 minimum wages	50.7	0.94 (0.87–1.02)		0.95 (0.87–1.02)			
Over 6 minimum wages	41.3	1		1			
Type of service used in last dental appointment			0.048				
Public	64.6	1.26 (1.02–1.54)					
Private	51.5	1					
Status at last dental appointment			0.001		0.002		
Very bad/Bad/Regular	78.9	1.54 (1.28–1.86)		0.83 (0.74–0.93)			
Very good/Good	51.3	1		1			
Block 3-Need Factors							
Self-perceived oral health			< 0.001		< 0,001		0.021
Bad/Regular	81.0	1.63 (1.39–1.91)		0.83 (0.75–0.91)		0.86 (0.76–0.98)	
Excellent/Good/Very good	49.6	1		1		1	
Satisfaction with appearance of teeth and mouth			0.005				
Very unhappy/Unhappy/Neither happy nor unhappy	61.1	1.28 (1.08–1.51)					
Very happy/Happy	47.8	1					
Toothaches over the last 2 years			< 0,001		0.002		0.028
Yes	64.3	1.42 (1.20–1.68)		0.90 (0.85–0.96)		0.93 (0.87–0.99)	
No	45.2	1		1		1	
Self-perceived need for dental treatment			0.002				
Yes	59.4	1.32 (1.10–1.59)					
No	44.8	1					

*PR* Prevalence ratio, *95%CI* Confidence interval

^a^Pearson’s chi-square test

^b^Poisson regression with robust variance

## Discussion

The prevalence of recent non-use of dental services among university students in the present study was of 19.5%, a finding lower than what has been observed in population-based epidemiological surveys. In the last epidemiological survey on dental health, SB Brasil 2010, the prevalence of non-use of dental health services in the last 2 years among young people aged 15 to 19 years old in the Southeast region of the country was of 70.2% [20]; in the 2019 National Health Survey, the non-use of dental services in the year prior to the interview by adults aged over 18 years, was at 46.8% [21]. However, it should be noted that these surveys, despite including a part of the young population, do not cover the specific age group selected in this study. In addition, no studies were found in the literature on the recent use of dental services by university students, which makes this comparison difficult.

In the adjusted analysis, the type of service used and self-perceived need for dental treatment were associated with recent non-use of dental services. Young people who used private services and who did not perceive a need for treatment were more likely to not use recent dental services. The importance of income as a determinant of access to dental services is already widely known in the literature, and is possibly aggravated in the young population, which has historically been disregarded by the public sector in defining priorities for oral health care [22]. The lack of perception of the need for treatment has also been indicated in the literature as one of the main reasons for not seeking dental care [23, 24]. Corroborating this non-perception is the fact that some oral diseases are asymptomatic at the beginning of their course, being identified by the individual only later [25]. Moreover, the perception of an individual feeling sick comes, besides the physical sensations of pain and discomfort, also from the social and psychological consequences that the evolution of oral diseases can cause. In both situations, the perception of the need for treatment and, consequently, the use of dental services may occur, many times, late and for curative purposes.

Despite the low prevalence of recent non-use of oral health services found in the present study, when the non-use of dental services on a regular basis by the population studied was assessed, a significant increase in this prevalence was observed, reaching 53.5%. This finding corroborates the limited national literature on the subject. A population-based study carried out by Carreiro et al. [7], in Minas Gerais, found a prevalence of 64.2% for regular non-use of dental services among individuals over 18 years of age. Echeverria et al. [8] assessed the regular use of dental health services by university students over 18 years of age in Pelotas (RS), finding a prevalence of 55%of non-use of these services, even closer to that found in this research.

It is important to highlight that the sample of this study is composed of college students and, despite of the university inclusion policies adopted in recent years, our study does not reflect the Brazilian population profile in this age group, because it does not include populations with greater social vulnerability, which have the greatest oral health problems and have less access to health services [8]. Moreover, it is noteworthy that regular use was obtained through self-report, which may generate an information bias.

The self-reported regular use of dental services by undergraduate students was associated with regular use of services during childhood, negative self-perception of oral health, toothache in the last 2 years, reason for the last dental visit, level of paternal education and area of knowledge of the course.

The habit of visiting the dentist in childhood showed association with a higher prevalence of not using it regularly. On the one hand, it is believed that if children develop patterns of regular dental care, they are likely to maintain this habit in adulthood [26]. However, access to dental care in youth may be hindered for various reasons, ranging from lack of maturity to seek health care, characteristic of age, to financial reasons related to the ability to pay for services. Brazil is going through a complex scenario both from the economic and political point of view, with direct repercussions on the health sector. There is evidence of a decrease in the number of individuals who can afford private services and, consequently, an increase in the number of patients who depend on public services; however, the financing of oral health policies has not followed this increase [27]. It should also be considered the fact that public dental services in Brazil have historically been offered in a priority manner to children of school age [28], and this privilege occurred to the detriment of care to other population segments [29]. To date, there is a gap in oral health policies and programs in relation to the young population. In this sense, it is possible that an individual who has had access to dental services in childhood, will have more difficulty in access to these services in youth, highlighting the need for actions aimed at this population.

Regarding the factors of need, the adjusted analysis revealed that young college students with negative self-perception of oral health and toothache in the last 2 years were less likely to not use dental services regularly. Self-perception of oral health measures the value placed on oral health and determines the likelihood of seeking care with the goal of achieving optimal oral health status [30]. Thus, both negative self-perception of oral health and recent experience of pain may result in the individual’s perceived need for treatment and, consequently, influence care-seeking.

Students who used services for symptomatic or curative reasons at their last visit were less likely to not use dental services regularly. This result seems to show that even those students who claim to use services even in the absence of problems are still more motivated by curative reasons. Echeverria et al. [8] also found that university students in southern Brazil used the service more to solve oral health problems and not on a regular basis to prevent aggravations. Other studies have demonstrated limited knowledge of oral hygiene practices and seeking dental care only in case of severe pain or discomfort by university students from various fields [31, 32].

The latter two associations were contrary to what was expected by the evidence already existing in the literature [2, 3, 33]. Young people whose parents had lower levels of education were less likely to not use regularly. Moreover, students from health or biological sciences courses presented higher probability of not using regularly when compared to undergraduates from other fields of knowledge.

As already mentioned, an important limitation of the present study concerns the collection of self-reported information on the regularity of dental visits. Considering that this is a socially desirable behavior, it is likely that there was an overestimation of the report of regular dental visits for preventive reasons, causing an information bias. Further studies on the regular use of dental services by this population with the use of objective indicators are suggested in order to minimize information bias.

Other limitations include the study design (since being a cross-sectional study cause and effect relationships cannot be determined) and losses in the sample, which may have been enhanced by the use of the online questionnaire and email contact. In addition, it should be considered that the motivation to participate in the study can influence the response patterns. For example, individuals who attach greater importance to oral health may feel more motivated to participate in the study; on the other hand, individuals with greater need for treatment may also be more interested in participating in the study. Thus, some parameters may have been over- or under-estimated.

Despite the limitations, this study contributes by broadening the focus on a population still poorly investigated in the literature and lacking oral health public policies that understand their specificities. Furthermore, we highlight the verification of two different patterns of use of dental services by young university students.

This evidence points to the need to develop programs and actions aimed at young university students, who have been neglected within the scope of oral health policies, in order to break the cycle of using dental services for curative treatments or for emergency care.

The data described in this article can be freely and openly accessed at figshare: 10.6084/m9.figshare.20980357.v2 [34].

## Conclusion

It was concluded that young university students use dental services motivated by curative treatment needs and not with a preventive purpose, as would be the ideal. Thus, public policies for the prevention and promotion of oral health in higher education institutions must be planned and implemented, as well as expanding access to public dental services to the young adult population, in order to guarantee improvements in the quality of life of this population.

## Data Availability

The datasets generated and analysed during the current study are available in the figshare repository, 10.6084/m9.figshare.20980357.v2.

## Abbreviations

UFJF: Universidade Federal de Juiz de Fora
MG: Minas Gerais
SPSS: Statistical Package for the Social Sciences
